# Comparison of acupuncture and pinaverium bromide in the treatment of irritable bowel syndrome: A protocol for systematic review and meta-analysis

**DOI:** 10.1097/MD.0000000000025604

**Published:** 2021-04-23

**Authors:** Huaiyu Li, Yun Chen, Ziyi Hu, Ying Yi, Jing Ye, Yuliang Zhou, Zhiying Yu, Haiyi Tang

**Affiliations:** a Jiangxi University of Traditional Chinese Medicine, Nanchang; b First Affiliated Hospital of Gannan Medical University, Ganzhou; c The Affiliated Hospital of Jiangxi University of Traditional Chinese Medicine, Nanchang, China.

**Keywords:** acupuncture, complementary therapy, functional gastrointestinal diseases, irritable bowel syndrome, pinaverium bromide

## Abstract

**Background:**

Irritable bowel syndrome (IBS) is one of the most common chronic gastrointestinal diseases, and the current diagnosis of IBS is still based on symptoms and examination. Pinaverium bromide is commonly used as an antispasmodic in the treatment of IBS. But adverse effects of pinaverium bromide are common. Meta-analyses show that acupuncture has a positive therapeutic effect on IBS.

**Methods:**

Randomized controlled trials of comparing the efficacy of acupuncture and pinaverium bromide in the treatment of IBS will be searched in the relevant database: PubMed, Embase, Cochrane Library, China National Knowledge Infrastructure (CNKI), Wanfang Database, Chinese Biomedical Literature Database (CBM), and Chinese Scientific Journal Database (VIP database). The studies selected will be exported to EndNote V.9.1 software. Data will be carried out independently from the selected articles by 2 reviewers. Any disagreement will be solved in consultation with a third reviewer.

**Results:**

Our study aims to compare the efficacy of acupuncture and pinaverium bromide in the treatment of IBS and to fill the lack of relevant evidence.

**Conclusion:**

Through the inclusion of relevant literature, the overall efficacy of acupuncture and pinaverium bromide in the treatment of IBS will be evaluated, and the gap between various acupuncture treatment measures will be further analyzed.

**INPLASY registration number:**

INPLASY 202130068.

## 1 Introduction

Traditionally, irritable bowel syndrome (IBS) is one of the most common chronic gastrointestinal diseases, and its pathophysiological mechanism is not clear.^[1]^ The main feature of IBS is recurrent abdominal pain, accompanied by abnormal stool shape or bowel habits.^[2]^ Due to the lack of relevant specific indicators, the current diagnosis of IBS is still based on symptoms and examination.^[3]^ The prevalence of IBS in most countries around the world is between 5% and 10%.^[4,5]^ Constipation predominant irritable bowel syndrome is more common in women, while diarrhea predominant irritable bowel syndrome is more common in men.^[6,7]^ The economic burden of IBS on Chinese patients and health care may account for 3.3% of the entire medical budget.^[8]^ Laxatives, antidiarrheal, and antispasmodics are all used as first-line treatment in IBS.^[9]^ Pinaverium bromide is commonly used as an antispasmodic in the treatment of IBS, and it can effectively relieve the pain caused by intestinal spasms and relieve stool problems in IBS patients.^[10,11]^ However, pinaverium bromide has several disadvantages: for acute abdominal pain in IBS, the number of times pinaverium bromide is taken is limited to a maximum of 3 times a day;^[12]^ adverse effects (AEs) of pinaverium bromide are more common, and many patients will experience symptoms such as dry mouth, dizziness, and blurred vision after taking it.^[13]^

As an indispensable part of Chinese medicine, acupuncture has become an indispensable part of global medical practice due to its efficacy and safety.^[14,15]^ Acupuncture is effective in the treatment of some functional gastrointestinal diseases,^[16]^ and it can significantly alleviate the symptoms of gastroesophageal reflux disease,^[17]^ functional dyspepsia,^[18]^ and inflammatory bowel disease.^[19]^ Several meta-analyses show that acupuncture also has a positive therapeutic effect on IBS.^[20–22]^ There have been some studies on IBS in China comparing pinaverium bromide with acupuncture, but few studies have evaluated the overall efficacy about it. People unable to obtain an overall understanding of the difference in the efficacy of the 2 treatments for IBS. Therefore, the purpose of our protocol is to select all randomized controlled trials (RCTs) related to acupuncture vs pinaverium bromide in the treatment of IBS, and to conduct quantitative or qualitative analysis to evaluate the effectiveness of acupuncture vs pinaverium bromide in the treatment of IBS. The comprehensive curative effect and the curative effect comparison between different acupuncture intervention methods.

## 2 Objectives

The aims are:

1. to comparing the efficacy of acupuncture and pinaverium bromide in the treatment of IBS;
2. to analyze the effects of different acupuncture interventions on IBS.

## 3 Methods and analysis

### 3.1 Study registration

The protocol of our study is conducted in strict accordance with the PRISMA-P guidelines and the Cochrane Handbook.^[23,24]^ This protocol has been registered on INPLASY (registration number: INPLASY 202130068: https://inplasy.com/inplasy-2021-3-0068/).

### 3.2 Inclusion criteria

#### 3.2.1 Type of studies

All RCTs which compared acupuncture with pinaverium bromide. RCTs conducted in adults without regional and language restrictions.

#### 3.2.2 Type of participants

All patients diagnosed with IBS, regardless the age, sex, source of cases, and IBS type. Diagnosis of IBS based on specific diagnostic criteria (Rome I criteria, Rome II criteria, Rome III criteria, Rome IV criteria, or the Manning criteria).

#### 3.2.3 Type of interventions

The intervention group is defined as acupuncture treatment, such as electroacupuncture, warm acupuncture, moxibustion, ear acupuncture, fire needling, or elongated needle. The acupoint numbers, retaining time, and frequency will not be restricted in this protocol.

#### 3.2.4 Type of comparators

The control group that will include Patients with IBS taking pinaverium bromide.

#### 3.2.5 Types of outcome measures

##### 3.2.5.1 Primary outcomes

The primary outcomes assessed will be the total effective rate.

##### 3.2.5.2 Secondary outcomes

Secondary outcome measures include the IBS Symptoms Severity Score, the IBS Quality of Life, 36-Item Short Form, and the rate of AEs.

### 3.3 Exclusion criteria

1. Nonrandomized controlled trials;
2. None of the valid outcome indicators;
3. None of the outcome indicators for this study;
4. Unfinished protocol;
5. Animal experiment, review and cohort studies will be excluded.

### 3.4 Search methods for identification of studies

#### 3.4.1 Electronic searches

The following databases will be searched: PubMed, Embase, Cochrane Library, China National Knowledge Infrastructure (CNKI), Wanfang Database, Chinese Biomedical Literature Database (CBM) and Chinese Scientific Journal Database (VIP database). The key words include “acupuncture,” “electro-acupuncture,” “auricular point,” “warm acupuncture,” “irritable bowel syndrome,” “IBS,” “pinaverium,” “pinaverium bromide,” “Dicetel.” An equivalent translation of the same search terms will be used to search in the Chinese databases. A combination of free words and medical subject headings terms will be used for searching. The searching strategy of PubMed is presented in Table 1.

**Table 1:** Search strategy used in PubMed database.

Order	Search items
#1	((((((((((Irritable bowel syndrome[MeSH Terms])) OR (Irritable Bowel Syndromes)) OR (Syndrome, Irritable Bowel)) OR (Syndromes, Irritable Bowel)) OR (Colon, Irritable)) OR (Irritable Colon)) OR (Colitis, Mucous)) OR (Colitides, Mucous)) OR (Mucous Colitides)) OR (Mucous Colitis)
#2	(((((((((((((((((Acupuncture[MeSH Terms]) OR (Pharmacopuncture)) OR (Acupuncture Therapy)) OR (Electroacupuncture)) OR (Manual Acupuncture)) OR (Dry Needle)) OR ((Moxibustion[MeSH Terms]) OR (moxibustion))) OR (Acupuncture, Ear[MeSH Terms])) OR (acupuncture, Ear)) OR (ear acupuncture)) OR (Auricular Acupuncture)) OR (Ear Acupuncture)) OR (Acupuncture, Auricular)) OR (acupuncture, Auricular)) OR (auricular acupuncture)) OR (Warm Acupuncture)) OR (Fire Needling)) OR (Elongated Needle)
#3	randomized controlled trial[Publication Type] OR randomized[Title/Abstract] OR placebo[Title/Abstract]
#4	#1 AND #2 AND #3

#### 3.4.2 Searching other resources

We will search the National Institutes of Health clinical registry Clinical Trials, International Clinical Trials Registry Platform, and ClinicalTrials.gov to find any ongoing or unpublished trial.

### 3.5 Selection of studies

**Figure 1:**
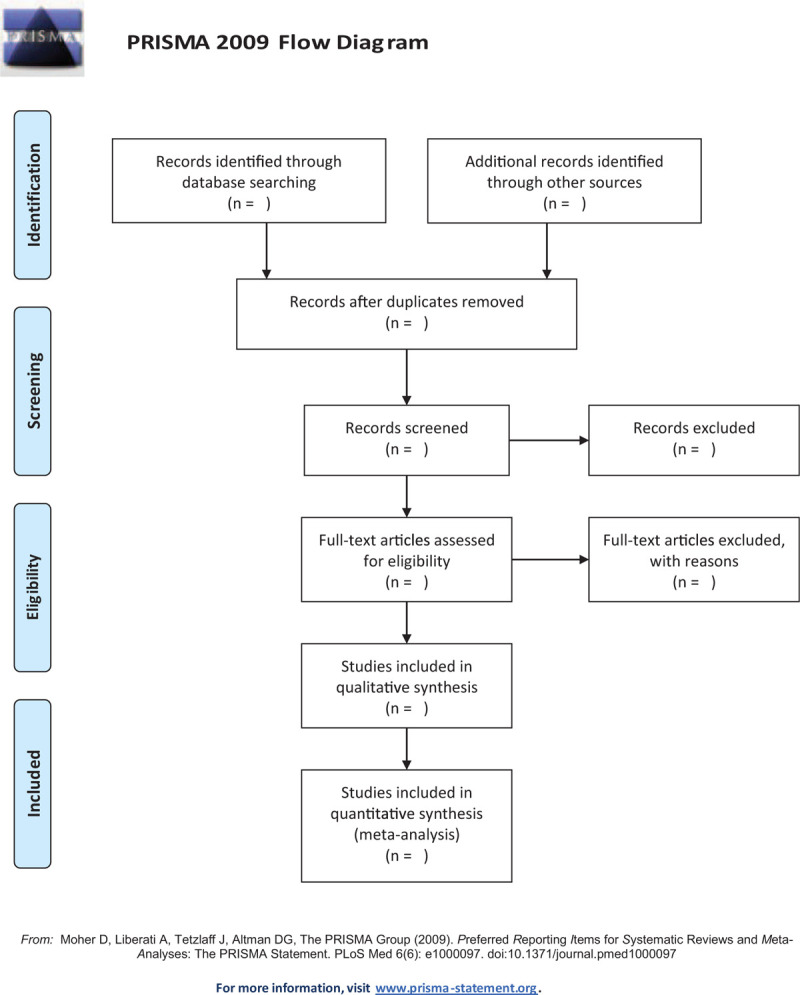
Flowchart of literature selection.

The studies of electronic searches will be exported to EndNote V.9.1 software to remove duplicate literature. Literature screening will be conducted independently by 2 reviewers according to the inclusion and exclusion criteria. They first read the title and abstract of the studies to exclude irrelevant trials. Then they read the full text to further screen out the documents that meet the requirements. Finally, valid data will be extracted in the included literature one by one. If there is a disagreement during the screening process, the final decision will be reached through discussion with the third reviewer. The selection process will be showed in a PRISMA flow diagram (Fig. 1).

### 3.6 Data extraction and management

Data will be carried out independently from the selected articles by 2 reviewers using a Microsoft Excel spreadsheet. Information extracted from each included article will include first author, publication year, sample size, characteristics of participants, type of treatments, outcome measures, and adverse events. The causes of both selections will be documented and full texts will be obtained and checked for further evaluation if necessary. We will try to contact corresponding authors for missing data. If the missing data cannot be obtained, we will delete the studies related to the missing data.

### 3.7 Assessment of the methodological quality

The risk of bias in the included literature will be assessed according to the Cochrane Collaboration's tool for assessing risk of bias.^[24]^ We will assess the risk of bias from the following 7 items: random sequence generation, allocation concealment, blinding of participants and personnel, blinding of outcome assessment, incomplete outcome data, selective reporting and other sources of bias. The risk of bias graph and the risk of bias summary will be generated by Review Manager (RevMan) V.5.3 software. Any disagreement should be solved in consultation with a third reviewer.

### 3.8 Measures of treatment effect

The relative risk will be used to assess dichotomous outcomes and weighted mean difference or standardized mean difference will be used in the analysis of continuous outcomes. 95% of the confidence intervals of dichotomous data and the continuous data will be determined in pooled estimates.

### 3.9 Dealing with missing data

We will attempt to contact authors to obtain missing data. If we cannot contact the original authors, the studies will be excluded from the data synthesis.

### 3.10 Assessment of heterogeneity

The Cochran Q statistics will be employed to assess heterogeneity. The results of the *I*^2^ statistic, which determine the using of fixed-effects model or random-effects model, cover unimportant heterogeneity (0%–40%), moderate heterogeneity (30%–60%), substantial heterogeneity (50%–90%), and considerable heterogeneity (75%–100%). If there exists significant clinical heterogeneity or methodological heterogeneity, a random-effect model or subgroup analysis will be performed to explore sources of heterogeneity.

### 3.11 Data synthesis

Fixed effects models will be used if the I^2^ value is <50%. Otherwise, we will remove low-quality studies and use sensitivity analysis to investigate which study has the most significant impact on heterogeneity. If quantitative synthesis is not possible, we will make a qualitative description.

### 3.12 Subgroup analysis

If there is significant heterogeneity between the study results, we will perform a subgroup analysis to investigate differences in gender, age, types of acupuncture interventions styles, etc.

### 3.13 Sensitivity analysis

We will use sensitivity analysis to evaluate the stability of decision-making during the review process. Several factors in the meta-analysis process will be taken into consideration, such as low-quality research, small sample research, etc. In addition, we will give the results of the sensitivity analysis in the summary table. The results of the sensitivity analysis will discuss the risk of bias in the meta-analysis.

### 3.14 Grading the quality of evidence

Two reviewers will independently use the Grading of Recommendations Assessment, Development and Evaluation (GRADE). According to the grading standard, the quality of evidence is graded as high, medium, low, or very low.^[25]^

### 3.15 Ethics and dissemination

The study will be published in a peer-reviewed journal or relevant conference. No ethical approval is required. The results of the study will provide potential evidence in advancing the therapeutic strategy of patients with IBS.

## 4 Discussion

In view of the current lack of understanding of the pathophysiology of IBS, clinical treatment strategies for IBS focus on the improvement of symptoms. A study shows that only nearly one-third of IBS patients in the United States are satisfied with the results of their treatment.^[26]^ In addition, many patients have stopped taking the medication because their symptoms have not improved significantly.^[27]^ Dissatisfaction with treatment and drug AEs have led patients to seek help from acupuncture, which is effective in the treatment of gastrointestinal disorders. Although there are some original literatures on the treatment of IBS with acupuncture, there are still very few meta-analyses to evaluate the efficacy of acupuncture and pinaverium bromide in the treatment of IBS. We will use the data of all relevant RCTs to conduct systematic reviews or meta-analysis to fill the lack of relevant evidence.

## Author contributions

**Conceptualization:** Huaiyu Li, Jing Ye.

**Data curation:** Huaiyu Li, Ying Yi, Haiyi Tang.

**Formal analysis:** Yun Chen, Yuliang Zhou, Zhiying Yu.

**Methodology:** Yun Chen, Ziyi Hu, Haiyi Tang.

**Software:** Ying Yi, Yuliang Zhou, Zhiying Yu.

**Supervision:** Ying Yi, Jing Ye.

**Writing – original draft:** Huaiyu Li, Ying Yi, Haiyi Tang.

**Writing – review & editing:** Jing Ye, Yuliang Zhou, Zhiying Yu.
